# Modelling strategies to break transmission of lymphatic filariasis - aggregation, adherence and vector competence greatly alter elimination

**DOI:** 10.1186/s13071-015-1152-3

**Published:** 2015-10-22

**Authors:** M. A. Irvine, L. J. Reimer, S. M. Njenga, S. Gunawardena, L. Kelly-Hope, M. Bockarie, T. D. Hollingsworth

**Affiliations:** School of Life Sciences, University of Warwick, Gibbet Hill Road, CV4 7AL Coventry, UK; Mathematics Institute, University of Warwick, Gibbet Hill Road, CV4 7AL Coventry, UK; Liverpool School of Tropical Medicine, Pembroke Place, Liverpool, L3 5QA UK; Faculty of Medicine, University of Colombo, Colombo, Sri Lanka; Kenya Medical Research Institute (KEMRI), P.O. Box 54840, 00200 Nairobi, Kenya

## Abstract

**Background:**

With ambitious targets to eliminate lymphatic filariasis over the coming years, there is a need to identify optimal strategies to achieve them in areas with different baseline prevalence and stages of control. Modelling can assist in identifying what data should be collected and what strategies are best for which scenarios.

**Methods:**

We develop a new individual-based, stochastic mathematical model of the transmission of lymphatic filariasis. We validate the model by fitting to a first time point and predicting future timepoints from surveillance data in Kenya and Sri Lanka, which have different vectors and different stages of the control programme. We then simulate different treatment scenarios in low, medium and high transmission settings, comparing once yearly mass drug administration (MDA) with more frequent MDA and higher coverage. We investigate the potential impact that vector control, systematic non-compliance and different levels of aggregation have on the dynamics of transmission and control.

**Results:**

In all settings, increasing coverage from 65 to 80 % has a similar impact on control to treating twice a year at 65 % coverage, for fewer drug treatments being distributed. Vector control has a large impact, even at moderate levels. The extent of aggregation of parasite loads amongst a small portion of the population, which has been estimated to be highly variable in different settings, can undermine the success of a programme, particularly if high risk sub-communities are not accessing interventions.

**Conclusion:**

Even moderate levels of vector control have a large impact both on the reduction in prevalence and the maintenance of gains made during MDA, even when parasite loads are highly aggregated, and use of vector control is at moderate levels. For the same prevalence, differences in aggregation and adherence can result in very different dynamics. The novel analysis of a small amount of surveillance data and resulting simulations highlight the need for more individual level data to be analysed to effectively tailor programmes in the drive for elimination.

**Electronic supplementary material:**

The online version of this article (doi:10.1186/s13071-015-1152-3) contains supplementary material, which is available to authorized users.

## Background

Lymphatic filariasis (LF) is one of the neglected tropical diseases (NTDs) targeted for local and global elimination as a public health problem within the next decade. The disease is caused by one of a group a mosquito-borne filarial nematodes (*Wuchereria bancrofti* (responsible for 90 % of cases), *Brugia malayi* or *Brugia timori*) and can lead to chronic morbidity, such as lymphedema, which is associated with pain, severe disability and resulting social stigmatisation.

It has been estimated that 1.24 billion people are at risk of LF in tropical and sub-tropical countries in Africa, Asia, the Western Pacific, the Caribbean and South America [1, 2]. In response to the large scale prevalence internationally and the potential to eradicate, the WHO launched the Global Programme to Eliminate Lymphatic Filariasis (GPELF) in 2000. The current aim of GPELF is to use mass drug administration (MDA) with a combination of donated drugs to reduce and eventually break transmission. In areas co-endemic with onchocerciasis, the combination of drugs used in MDA are ivermectin and albendazole (ALB), whereas diethylcarbamazine (DEC) and ALB are used in other endemic regions. The current strategy is to have yearly treatment at 65 % coverage for at least 5 years, followed by regular transmission assessments to identify whether transmission has been broken. Initiation of MDA programmes will be accompanied by morbidity management. The World Health Organisation has set the target of eliminating lymphatic filariasis as a public health problem globally by 2020 [3]. A number of countries have reached the targets of transmission interruption or MDA termination and others have scaled up their treatment programmes and are getting close to these targets, reducing the risk of infection for hundreds of millions of people. However, there are still large numbers of affected populations who are unlikely to receive the minimum 5 rounds of treatment by 2020. For these countries the most effective way to meet the target, if it can be met, is still open to discussion. Alternate strategies include (i) MDA at high coverage, (ii) adding moderate vector control or (iii) twice-yearly treatment.

Mathematical modelling has played a part in understanding the transmission dynamics of lymphatic filariasis for many decades (see review by Stolk et al. [4]). These models have shown that both reductions in mosquito biting rates and MDA have the potential to reduce and even break transmission of LF, but have also highlighted the importance of ensuring transmission has truly been broken and maintaining vector control to prevent bounce-back of infection. There have been some estimates of the number of rounds of treatment required to break transmission, for example, Stolk et al. [5] suggest MDA programmes may be required for more years in Africa than in India due to the higher efficacy of DEC + ALB. They also highlighted that increasing coverage is likely to be an equally effective strategy than treating twice a year, with the suitability of each depending on local implementation costs [5]. There has also been some discussion of the importance of parameter uncertainty, for example Gambhir et al. [6] focused on the importance of such uncertainty on the predictions of breakpoints (threshold parasite densities at which transmission cannot be maintained). The two main models, EPIFIL [7], a deterministic model, and LYMFASIM [8], an individual based model, have been fitted to a limited number of datasets, primarly the Pondicherry study [5, 6, 9, 10] and cross-sectional data, such as that from Tanzania and Kenya [9]. Different modelling papers give very different estimates of the number of rounds required to achieve elimination in different settings, with few papers giving a systematic overview of the drivers for these differences, and very few models validated against surveillance data from multiple settings [4]. There is also a need to validate models against surveillance data and combine it with coverage data in order to make projections to help programmes in breaking transmission [11].

Here we develop a closely related model to both EPIFIL and LYMFASIM, but fit and validate it against recent individual-level surveillance datasets for two quite different MDA programmes in Kenya and Sri Lanka. We then simulate the model across a range of different settings to see how yearly MDA compares with (i) MDA at high coverage, (ii) adding moderate vector control or with (iii) twice-yearly treatment in order to study how these affect the probability of elimination after 5 years. We investigate the role of the main transmitting vector, the level of aggregation of worms amongst people in the population and patterns of non-compliance with the treatment programme on these results. The aim of the analysis is to understand how simulation outcomes are dependent on factors associated with setting. They also will identify the extent to which control programmes need to be informed by local epidemiological data, such as prevalence and the level of aggregation of worms, or the likely coverage of treatment and vector control programmes.

## Methods

### Model

We introduce a novel model of filariasis infection in humans, which can broadly be described as a stochastic equivalent to the deterministic EPIFIL model without immunity. A full model description is given in the Additional files, however we shall briefly give a summary of the model development here. The model is a stochastic micro-simulation of individuals with worm burden, microfilaraemia and other demographic parameters relating to age and risk of exposure. Humans are modelled individually, with their own male and female worm burden. The concentration of mf in the peripheral blood is modelled for each individual deterministically and increases according to the number of fertile female worms as well as decreasing at constant rate. The total mf density in the population contributes towards the current density of L3 larvae in the human-biting mosquito population, where the distribution of L3 amongst the human-biting mosquito population is completely homogeneous. An empirically derived relationship is used for the uptake of mf by a mosquito, where both *Culex* and *Anopheles* uptake curves are implemented depending on setting. The model dynamics are therefore divided into the individual human dynamics, including age and turnover; worm dynamics inside the host; microfilariae dynamics inside the host and larvae dynamics inside the mosquito.

### MDA intervention

The effect of MDA was simulated for an individual by reducing their mf load and worm numbers according to estimated drug efficacies from the literature (See Table 1 for a summary of these efficacies and the corresponding references). After the MDA intervention the dynamics continue as normal and there is no lasting effects of MDA except for the initial reduction in mf and worm number.

**Table 1:** Summary of parameter values used to inform the model

Parameter symbol	Definition	Value	Source
*n*	Size of population in simulation	1000 unless otherwise stated	N/A
*λ*	Number of bites per mosquito	10 per month	[37, 38]
*V*/*H*	Ratio of number of vectors to hosts	Fitted to data	N/A
*α*_max_	Age at which exposure to mosquitoes reaches its maximum level	20.0	[12]
*ψ*_1_	Proportion of L3 leaving mosquito per bite	0.414	[39]
*ψ*_2_	Proportion of L3 leaving mosquito that enter host	0.32	[40]
*s*_2_	Proportion of L3 entering host that develop into adult worms	0.00275	[7, 41]
*μ*	Death rate of adult worms	0.0104 per month	[42]
*α*	Production rate of Mf per worm	0.2 per month	[39]
*γ*	Death rate of Mf	0.1 per month	[39, 43]
*ɡ*	Proportion of mosquitoes which pick up infection when biting an infected host	0.37	[45]
*σ*	Death rate of mosquitoes	5 per month	[40]
*k*	Aggregation parameter of individual exposure to mosquitoes	Fitted to data	[23, 24]
*h*(*a*)	Parameter to adjust rate at which individuals of age α are bitten	Linear from 0 to 10, with maximum of 1	[7]
*χ*_1_	Proportion of Mf killed for an individual MDA round using ALB & DEC	0.95	[46, 47]
*κ*_1_	Proportion of adult worm permanently sterlised during MDA round using ALB & DEC	0.55	[46, 47]
*ρ*	Systematic adherence of MDA	Varied	N/A
*p*_*C*_	Coverage of MDA	Varied	N/A
*p*_*N*_	Coverage of LLIN	Varied	N/A
*ρ*_*BC*_	Correlation between bite exposure and adherence to MDA	Varied	N/A
*ρ*_*CN*_	Correlation between adherence to MDA & LLIN	Varied	N/A

### Simulation outputs

In order to compare simulations to data a number of key epidemic statistics were defined using the outputs from a simulation. The probability that an individual is mf positive is given as the Poisson probability of a positive number of mf being detected with rate taken as the individual’s current mf load. The ICT prevalence was calculated by averaging over all individuals who have a positive number of worms. The mf intensity distribution was calculated using the mf load values for each individual at a given time-point.

### Endemic prevalence

**Fig. 1:**
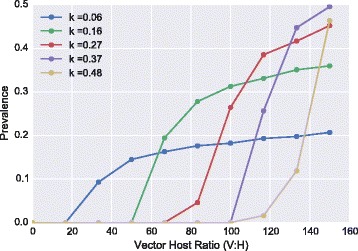
Prevalence to vector-host ratio relationships. The endemic prevalence for a range of vector to host ratios at various population heterogeneity levels, k. Threshold behaviour is more pronounced when the population is less aggregated. When the population is more aggregated, there is little threshold behaviour. With the lowest k, corresponding with the highest level of aggregation, there is a smooth transition from zero prevalence to a positive prevalence within the population

The model parameters that were varied according to the setting were the aggregation of bite exposure and the vector to host ratio. The endemic prevalence of mf is affected by both of these setting parameters (Fig. 1). There is a threshold behaviour where the endemic prevalence increases sharply from zero as the vector to host ratio increases. This threshold is highly dependent on the aggregation of the bite risk within the population controlled by the parameter *k*. A smaller *k* (more aggregated population) leads to a non-zero endemic equilibrium at lower vector to host ratios than are observed when the bite-rate is more homogeneous. This is a consequence of when the bite risk is more aggregated, there are individuals who are more highly burdened that drive the infection above the breakpoint threshold. When the population is more aggregated the endemic prevalence can occur at lower levels than when the population is more homogeneous.

### Data

The model was fitted and validated against two studies of an MDA programme over a number of years. The first is a study of two rounds of MDA using DEC combined with ALB across four sentinel communities in Malindi, Kenya in the years 2002–2004 [15]. Baseline blood samples were taken in all four communities before the first round of MDA in February 2002. Thereafter, blood samples were taken in March 2003 (post-MDA1) and July 2004 (post-MDA2). Both the immune-chromatographic antigen (ICT) and mf tests were performed on the same blood samples to provide an estimate for the prevalence of antigenaemia and microfilaraemia in the population. In order to retain statistical power, the results from the four communities were aggregated to provide a total number sampled in each survey of *n* = 808.

Further model fitting was performed on a 2-year study in the districts of Colombo and Gampaha in Western Sri Lanka during an MDA programme [16]. This represents a contrasting dataset as the dominant vector species is *Culex*, but where the main drug combination used in MDA is still ALB and DEC. Antigenaemia was measured through ICT and microfilaraemia measured through membrane filtration. Measurements were taken after an MDA round in August 2004 and just prior to another MDA round in late July 2005.

### Model fitting & comparison

The model was fitted using a maximum likelihood framework. The expected age-prevalence profile was calculated for a set of parameters by averaging across 1000 simulation runs. A likelihood was then constructed assuming binomial sample error for the number of individuals testing positive in each age category of the total population of individuals surveyed. For the Kenyan dataset, the initial time-point was at baseline before intervention. The age-prevalence curve was recorded from the model after reaching endemic equilibrium and used in the model fitting. For the Sri Lankan dataset, the initial time-point used in the model fitting was after the first round of MDA. In order to perform fitting on this dataset, the simulation was run until equilibrium at which point an initial MDA round was simulated. The age-prevalence profile was then taken 2 months after the initial round and used to compute the likelihood.

The fitted simulation was then compared to the subsequent time-points in each dataset for both the mf and ICT prevalence. MDA was simulated for coverage taken from the data with efficacy taken from literature estimates. Alongside this the mf intensity distribution was also compared at baseline for the Kenyan dataset. This was not done for the Sri Lankan dataset as no baseline measurements were taken and the number of non-zero mf counts were too few.

### Scenarios

A number of scenarios are considered that can impact the effectiveness and timeline of a MDA programme. These are where systematic adherence of MDA occurs where individuals either consistently adhere or not adhere to the programme; where correlation exists between bite risk exposure and adherence; where different forms of vector controls occur alongside an MDA programme; and where correlation exists between the MDA and vector control programme (Table 1 provides a summary of the parameters used in these scenarios).

Five distinct intervention strategies were compared for the elimination of LF. These were mass drug administration at 65 % coverage, mass drug administration at a higher 80 % coverage, bi-annual MDA at 65 % coverage, annual MDA at 80 % coverage with use of LLINs for *Anopheles* and reduction of bite-rate by 50 % in *Culex* areas. These were compared in high, medium and low transmission settings. Table 2 provides a full list of the assumptions used in each scenario.

**Table 2:** Baseline intervention assumptions

Scenario	Species				
		Coverage (%)	Frequency (months)	Bite rate (%)	LLIN coverage (%)
1	*Anopheles*	65	12	100	0
	*Culex*	65	12	100	0
2	*Anopheles*	80	12	100	0
	*Culex*	65	12	100	0
3	*Anopheles*	65	6	100	0
	*Culex*	65	6	100	0
4	*Anopheles*	65	12	100	65
	*Culex*	65	12	50	0

### Systematic adherance

Population adherence has been identified as a key factor associated with the success of an MDA programme [17]. If adherence is systematic then there are a certain group of individuals who are more or less likely to adhere to an intervention compared to the average. This could lead to individuals with higher burden not being treated due to geographic or planning factors, which can have negative consequences for reaching elimination. Systematic adherence is modelled explicitly, allowing a range of scenarios to be explored where semi-systematic adherence is present [18]. The systematic adherence parameter *ρ* controls the correlation in adherence between MDA rounds. When *ρ* = 0, MDA is randomly assigned to individuals. When *ρ* = 1, an individual either always adheres to the intervention or never adheres. For values in between zero and one a proportion of individuals adheres randomly, whilst a person’s decision to adhere or not remains the same for each round. Details of the implementation are given in the Additional files.

### Correlation between systematic adherence and exposure risk

We also explore scenarios under which there may be a correlation between the bite-risk for an individual and the individual’s tendency to adhere or not adhere with a treatment. There can be both a positive-correlation, where the higher bite-risk is associated with a higher tendency to not adhere with mass drug administration or a negative correlation, where a higher bite-risk increases the likelihood of adhering with mass drug administration.

### Vector control

**Fig. 2:**
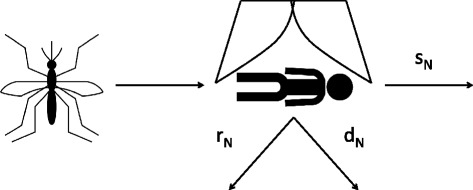
Schematic of mosquito interaction with LLINs. The diagram represents the interaction between the mosquito vector and LLINs, where there are three outcomes of biting event: repeating r_N_, death d_N_ or success s_N_

One of the established forms of vector control (VC) considered for filariasis is the use of Long-lasting insecticide-treated nets (LLINs). LLINs are considered for vector control in *Anopheles* settings and we adopt a similar modelling approach as in [19, 20]. Vector control increases the time between biting events and as a consequence decreases the bite rate as well as increasing the mortality. This change in the bite rate and mortality was modelled using the schematic in Fig. 2, where a mosquito attempting to feed where a bed net is present either repeats with probability *r*_*N*_, dies with probability *d*_*N*_ or is successful with probability *s*_*N*_.

The modified global bite rate based on coverage of bed nets (*p*_*N*_) is the original bite rate (*λ*) multiplied by a factor that determines a random bite being successful, *λ*(*p*_*N*_*s*_*N*_ + (1 ‐ *p*_*N*_)).

The increase in mortality is similarly calculated, however the mortality under intervention is the natural mortality (*σ*) added to the mortality probability due to the vector control (*d*_*N*_) and so is *σ* + *λp*_*N*_*d*_*N*_. It is assumed these effects are constant throughout the whole time the intervention is taking place.

An individual’s bite-rate is also affected based on their use of bed nets. This is calculated by reducing their bite-rate by a factor depending on whether the bite is successful due to the presence of LLIN. This is given by *λs*_*N*_ if the individual uses a bed net and *λ* otherwise (Table 3 provides a summary of these parameters including references). We conservatively assume coverage of bed-nets of 50 % when VC is present and do not include degradation effects of the nets or sporadic use.

**Table 3:** Summary of vector control parameters for *Anopheles gambiae s.s*

Parameter	Value	Description	Reference
*r*_*N*_	0.56	Repeating probability for LLINs	[48–50]
*s*_*N*_	0.03	Successful feeding with LLINs	[48–50]
*d*_*N*_	0.41	Insecticide mortality probability for LLINs	[48–50]

Vector control in a *Culex* setting was also modelled. Due to differences in feeding behaviour, other forms of vector control need to be considered. Polystyrene beads are used to cover pit latrines and other breeding sites for *Cx. quinquefasciatus* and have produced a large reduction in the annual bite-rate depending on the number of breeding sites that are covered [21]. We conservatively estimate a bite-rate reduction of 50 % when a *Culex* vector control programme is implemented. Although much higher reduction in bite rates have been observed (98 % [22]), the reduced reduction in bite rate was chosen to encompass a wider range of scenarios where VC may not be able to effectively cover all breeding sites.

## Results

### Fitting

The model was fitted to the Malindi, Kenya dataset using the maximum likelihood fitting procedure. The fitted parameters were the vector to human ratio *V*: *H* and the shape parameter corresponding to the heterogeneity of exposure risk among individuals, *k*. The maximum likelihood parameters were estimated to be *V*: *H* = 120 and *k* = 0.08.

**Fig. 3:**
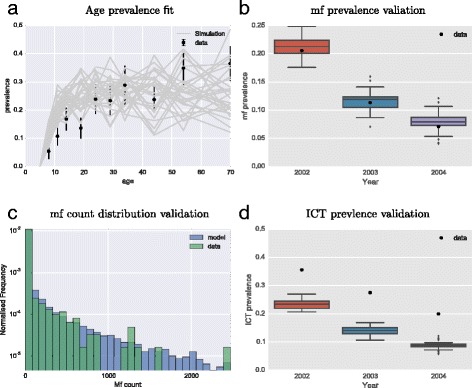
Fit and comparison of individual-based model to Malindi, Kenya dataset. The model was fitted using maximum likelihood to the baseline data in 2002. The MDA programme was modelled for the subsequent years and compared to the outcome from the data. **a** Baseline age prevalence data used in model fitting given as point estimates with standard errors. Example age prevalence curves from simulations shown in grey. **b** The range of prevalence from simulations are given as box-plots with the prevalence from the data given as a single point. **c** Comparison between model predicted mf-intensity distribution and mf-intensity distribution derived from data. Data colour is semi-transparent to show model distribution behind. **d** The range of prevalence from simulations are given as box-plots with the ICT prevalence from the data given as a single point

As a further step to compare the model fit to the data, the distribution of mf between individuals was compared between the recorded mf count data and the predicted distribution by the model (Fig. 3c). The distributions were compared for similarity using the Kolmogorov-Smirnov test statistic, which compares the maximum deviance between two empirically-derived cumulative distribution functions and is not rejected under standard hypothesis testing if the two distributions are statistically similar. This was performed by sub-sampling without replacement from the model distribution for the same number of individuals as in the data (*n* = 740). For 91 out of 100 re-samplings the Kolmogorov-Smirnov test statistic returned a p-value greater than 0.01 confirming that the two samples are drawn from the same underlying distribution. A visual comparison of the two distributions also confirms the similarity between the two distributions.

For further validation, the estimates of the ICT prevalence from the simulated MDA campaign were also compared to the data (Fig. 3d). The antigen prevalence is consistently under-estimated by the model for each year, although the decline in ICT is comparable to the decline observed, suggesting that the current model for ICT prevalence needs further validation.

**Fig. 4:**
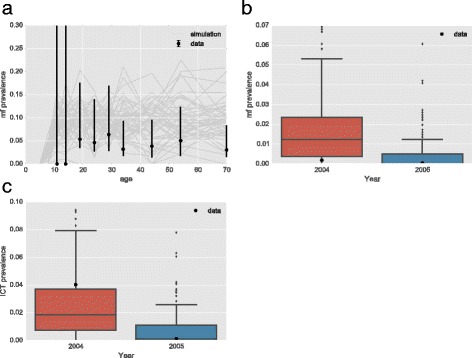
Fit and comparison of individual-based model to Sri Lanka dataset. **a** Age prevalence data used in model fitting given as point estimates with standard errors. Example age prevalence curves from simulations shown in grey. **b** The range of prevalence from simulations are given as box-plots with the prevalence from the data given as a single point. **c** The range of ICT prevalence from simulations given as box-plots with the prevalence from the data given as a single point

The fitting was also performed on the Sri Lankan dataset. Here the initial survey was taken while the area had already undergone MDA for a year. The parameters were once again fitted using maximum likelihood. The fitted parameters were found for vector to human ratio to be *V*: *H* = 55 and the population bite-risk aggregation *k =* 0.06. The resulting simulated age-prevalence profiles match reasonably well with the data and fall within the confidence intervals of all points, except for the first two age groups (Fig. 4a). The fit was then compared against the point-prevalence taken the following year after another round of MDA. The MDA programme was simulated with the same coverage and drug combination as in the survey. The simulations also match closely with the model for the following year (Fig. 4b).

The ICT prevalence was calculated from the number of individuals in a simulation at a single time-point with adult worms present (Fig. 4c). There is close agreement between the ICT prevalence simulated and the estimate in the data for both years with both estimates falling within the 95 % confidence intervals.

### Scenarios

Having fitted and compared the model against these contrasting datasets we now investigate the impact of different combinations of intervention strategies for a baseline case, for a scenario in which the heterogeneity of worms amongst people is much lower and for a scenario in which systematic non-adherence is considered.

### Baseline controls

**Fig. 5:**
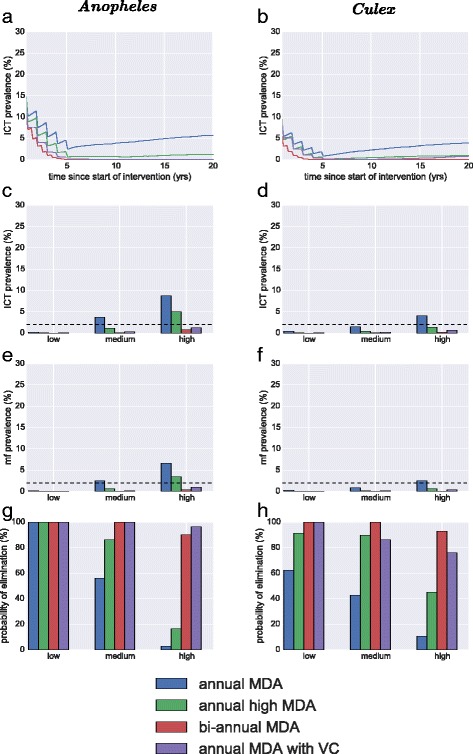
Comparison of main strategies for elimination in different settings. These are mass drug administration at 65 % coverage, mass drug administration at a higher 80 % coverage, bi-annual MDA at 65 % coverage and annual MDA at 80 % coverage. The three endemic settings considered are mf prevalence 15 % (high), 10 % (medium) & 5 % (low). The left column is in a setting where *Anopheles* dominates transmission and the right column is where *Culex* dominates transmission. **a & b** are the ICT prevalence for medium endemicity over 20 years averaged over the simulation runs. **c & d** are mf prevalence after 5 years; (**e**) & (**f**) are ICT prevalence after 5 years and (**g**) & (**h**) are the probability of elimination are 5 years

The five intervention strategies were compared assuming completely random coverage of MDA and vector control. We first present some example simulations of the averaged ICT prevalence over 200 simulations runs for a 20 year period from the start of intervention in a medium endemic setting (Fig. 5a, b). For the *Anopheles* setting (Fig. 5a) there is a steady decline for annual MDA, whereas more dramatic declines are observed for annual high coverage MDA, bi-annual MDA and MDA with VC. After 5 years, both bi-annual and MDA with VC do not increase as transmission has been broken in all runs. Annual MDA and annual high coverage MDA bounce back to a lower endemic level as some runs have achieved elimination whereas others have not. In the *Culex* setting, similar observations were made for the first 5 years of intervention (Fig. 5b). As these timelines are averaged of different runs, where some achieve elimination and others do not, the bounce back after 5 years is from those runs that have not achieved elimination. The probability of a run achieving elimination given that it fell below the 1 % threshold after 5 years was found to be one in all settings (not shown in figure).

To compare across scenarios we summarise the behaviour after 5 years of intervenions. Figure 5c & d show the antigen prevalence after 5 years of intervention for the scenarios in *Anopheles & Culex* settings respectively. In low transmission settings, greater reductions can be seen. For example in Anopheles settings (Fig. 5c) prevalence is below two percent (shown as a black dashed line) in all scenarios. The high endemic setting scenario provides the greatest contrast as bi-annual treatment and annual MDA with VC fall below 2 % and annual and annual high coverage MDA are above. In the medium transmission setting, only annaul MDA is above the threshold value. In the *Culex* setting, the ICT prevalence is only above the 2 % threshold for annual MDA in the high endemic setting (Fig. 5d), all the other scenarios are above the threshold after 5 years. Very similar observations are made for the mf prevalence (Fig. 5e, f) however they are noticeably reduced from the ICT.

Finally the probability of elimination is given for all scenarios in both the *Anopheles & Culex* setting (Fig. 5g, f). This shows the large discrepancy in outcomes of the scenarios in different endemic settings. For *Anopheles* (Fig. 5g) yearly MDA with VC is capable of achieving elimination in all settings, albeit at a slightly lower probability for the highest endemicity. The other interventions show a range of outcomes, with bi-annual MDA achieving elimination in low, medium and high settings. Annual high coverage of MDA can achieve elimination in low and medium transmission settings for 100 and 85 % of cases, but only 18 % in high transmission settings. Annual MDA only reliably leads to elimination in 5 years in the low endemic setting. In the *Culex* settings, the probability of elimination (Fig. 5h) is high in a low, medium and high transmission settings for bi-annual and MDA with VC. Annual high MDA achieves elimination in over 80 % of cases for low and medium settings, but less than half for high. Annual MDA is the worst performing, with 60, 43 and 9 % probability of elimination in low, medium and high settings.

### Heterogeneity of bite risk

Heterogeneity of bite risk amongst individuals, resulting in aggregation or clustering of worms within particular individuals, can vary between regions. In a less heterogeneous population interventions can be much more effective because the density of infective vectors necessary to sustain transmission is increased [23, 24]. This level of aggregation or clustering is different in different setting and can only be measured using the mf-intensity distribution. We investigated whether this variation was important by simulated the same scenarios as above, but for a much less clustered or aggregated worm population, but where the prevalence was matched to the baseline scenarios (5, 10 and 15 % mf prevalence). It is important to note that to achieve the same prevalence for a more homogeneous population the vector to human ratio will be lower, making control more achievable.

**Fig. 6:**
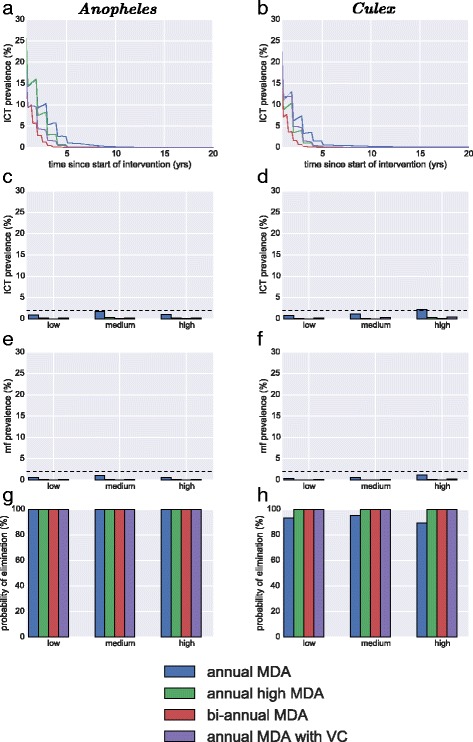
Comparison of main strategies for elimination of LF in different settings with a more homogeneous distribution of risk. These are mass drug administration at 65 % coverage, mass drug administration at a higher 80 % coverage, bi-annual MDA at 65 % coverage and annual MDA at 80 % coverage. The three endemic settings considered are mf prevalence 15 % (high), 10 % (medium) & 5 % (low). The left column is in a setting where Anopheles dominates transmission and the right column is where *Culex* dominates transmission. **a & b** are the ICT prevalence for medium endemicity over 20 years averaged over the simulation runs (**c**) & (**d**) are mf prevalence after 5 years; (**e**) & (**f**) are ICT prevalence after 5 years and (**g**) & (**h**) are the probability of elimination are 5 years

The resulting scenarios show a large difference in the outcome of interventions (Fig. 6). Figure 6a & b show the dramatic decrease in ICT prevalence for 20 years since the beginning of intervention. After 5 years transmission has been broken for all scenarios, with the more intensive scenarios such as bi-annual MDA achieving elimination before the 5 year target. Annual MDA also continues to decline after 5 years, although it’s reduction is slower than other scenarios only reducing the prevalence to near zero after 10 years. Both the ICT prevalence (Fig. 6c, d) and the mf prevalence (Fig. 6e, f) after 5 years indicate that all interventions have effectively broken transmission. This is confirmed by the probability of elimination for *Anopheles* and *Culex* (Fig. 6g, h) where there is 100 % elimination in all scenarios. The exception is with annual MDA in a Culex setting (Fig. 6h), however the probability is still above 80 % for all endemic settings.

### Adherence of intervention

**Fig. 7:**
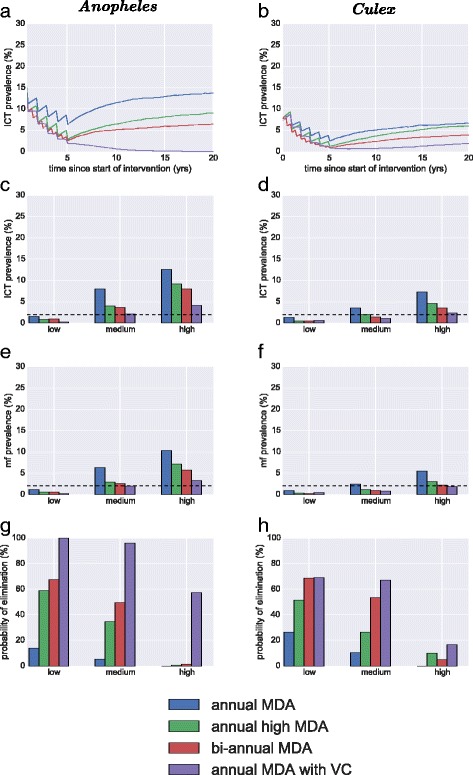
Comparison of main strategies for elimination of LF in different settings, with systematic adherence included. The interventions compared are mass drug administration at 65 % coverage, mass drug administration at a higher 80 % coverage, bi- annual MDA at 65 % coverage and annual MDA at 80 % coverage. The three endemic settings considered are mf prevalence 15 % (high), 10 % (medium) & 5 % (low). Furthermore, there is a 50 % correlation between whether an individual complies with the intervention in each round, combined with a correlation of 0.5 between bite exposure and MDA adherence as well as a 0.5 correlation between use of bed nets and adherence with MDA. The left column is in a setting where *Anopheles* dominates transmission and the right column is where *Culex* dominates transmission. **a & b** are the ICT prevalence for the medium endemic setting over 20 years averaged over the simulation runs; (**c**) & (**d**) are mf prevalence after 5 years; (**e**) & (**f**) are ICT prevalence after 5 years and (**g**) & (**h**) are the probability of elimination are 5 years

Another important factor which can affect the success of a programme, and an assumption which can have a large impact on model predictions, is the extent of systematic non-adherence in the population, and whether this is correlated with other factors. In the supplementary information we investigate these effects more extensively, but we summarise the results through the scenario analyses. The scenarios were investigated where on average 50 % of the population systematically complies with MDA. We also simulate a correlation coefficient of 0.5 between adherence with MDA and adherence with bed nets (reflecting a setting where access to all health care interventions is easier for some in the population than for others) and the same for bite risk and MDA (reflecting a scenario in which poor access to interventions is particularly acute for the high risk group). There is little information available on the realism, or otherwise, of this type of scenario, but we include it to illustrate the potential importance of these types of effects on the efficacy of a control programme. The results show that the effectiveness of these interventions can be undermined when these type of issues exist (Fig. 7). MDA with vector control is still likely to be able to achieve elimination in all cases for low and medium endemicity, but not in the high setting, whereas this was the case for the baseline scenario. The bi-annual intervention all suffered from a greatly reduced probability of elimination, declining from 100 to 65 %, Annual MDA at high coverage was able to achieve elimination 60 % of the time, with annual MDA only 14 %. The outcome for medium endemicity is also worsened, with most strategies ineffective at achieving elimination above 50 % with the exception of when vector control is included (Fig. 7g). In *Culex* settings the interventions in the face of these complications fare relatively similarly with a decline in elimination for all strategies and nearly no chance of elimination for annual MDA alone (Fig. 7h).

**Fig. 8:**
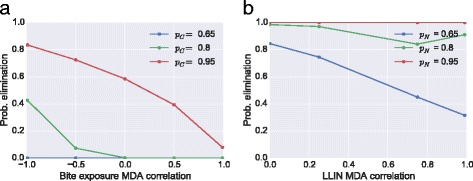
Impact of systematic correlations on MDA campaign. Correlation between adherence to MDA and other factors can negatively impact an elimination campaign. Probability of elimination after 5 years for an annual MDA programme at various MDA coverages (pC) and LLIN coverages (pN), where there is either (**a**) a negative to positive correlation with bite-risk and non-adherence to MDA or (**b**) a positive correlation between LLIN and MDA non-adherence

In order to investigate the impact of these types of correlations further, we look across a range of parameter values. When a higher bite exposure is associated with a tendency to not adhere to intervention decreases the success of an MDA programme, when a correlation exists between higher bite exposure and adherence, the probability of elimination can be dramatically increased. For example, annual MDA at 80 % coverage with high endemicity can increase the elimination probability from 2 to 48 % (Fig. 8a).

Correlation between MDA and LLIN adherence can also limit the success of an elimination campaign. For a moderate amount of systematic adherence and non-adherence (*ρ* = 0.75), a correlation between individuals who use LLIN and receive MDA treatment can dramatically decrease the probability of elimination. For example, with LLIN coverage of 65 %, a correlation can decrease the probability of elimination within 5 years from 80 to 30 % (Fig. 8b).

## Discussion

Elimination of lymphatic filariasis through the use of mass drug administration is a potentially complex problem with many open questions and challenges necessary to tackle as coverage increases globally. Nevertheless, the global programme has been very successful in a number of countries. However, where the programme is less successful it is difficult to disentangle the reasons for this failure, which may be multi-factorial. We have developed a novel model of filariasis infection amongst individuals with the primary aim of comparing strategies in terms of their likely impact on achieving elimination. The model builds upon two established models of LF infection, EPIFIL and LYMFASIM [7, 8]. It is able to reproduce aggregated infections observed across two settings as well as reproduce expected declines in both prevalence of mf and ICT. This new tool allows the comparison of a variety of intervention strategies used in combating filariasis, as well as giving the ability to model further complications in human behaviour and vector biology. We have compared these strategies with the aforementioned complications in order to provide robust estimates of the probability of elimination. The novelty of this analysis is the comparison of the model against longitudinal surveillance data from two quite different sites, in Kenya and Sri Lanka. The use of this longitudinal data gives confidence in the model’s predictions for mf prevalence, but highlights some remaining issues in terms of the simulation of ICT prevalence, the main tool for surveillance in recent years.

Simulations of the model suggest that MDA alone may result in elimination in many settings if coverage is sufficient or if a bi-annual treatment strategy is adopted. The timeline to elimination is broad and elimination within 5 years may only be possible when MDA is combined with vector control in certain high endemic settings. The simulations also suggest that when overall prevalence is below 2 % for mf and 1 % for ICT, recrudescence is unlikely to occur, although a formal analysis of this was not conducted here. Bi-annual MDA represents a significant increase in the resources and logistics required in order to achieve similar coverage with annual MDA [5]. Therefore, if annual intervention at higher coverage is able to achieve elimination rather than switching to bi-annual coverage, the former would be preferred. There may be an issue that annual MDA is not sufficient to break transmission and the prevalence will continuously bounce back between interventions. With such a case elimination may never be feasible within a reasonable timeline. Where the main vector of transmission is *Anopheles* annual MDA at 95 % coverage has similar outcomes to bi-annual coverage at 65 %. For a population of 1000, this means there are 350 fewer individual treatments required each year, along with significantly less resources and personnel cost. There is therefore evidence that increasing the coverage of an annual programme rather than switching to twice yearly would have the same results at reduced costs, which supports previous analysis [5]. A higher coverage however does represent a significant scaling up in terms of logistics and resources, for instance high coverage has been achieved for example in Sierra Leone during National Immunisation Days [25]. Accurate mapping combined with modelling can be an important tool in order to assess whether such a scale up of coverage is required.

One open challenge facing the estimates of elimination is knowledge of how heterogeneous the number of bites per individual is in a population where LF is endemic. A less heterogeneous bite risk means that an individual’s bite rate deviates little from the mean and hence epidemiological differences are fewer. This has a profound effect on an MDA programme as less difference between individuals means that random coverage of treatments is able to achieve elimination sooner [23, 24]. If the population is more heterogeneous then some individuals may have comparatively much higher worm burdens and mf compared to the average. This has two impacts: one is that the overall prevalence appears low as a disproportionate amount of burden is in a smaller number of the population and a random coverage of MDA is less effective as it may not cover those individuals who have high burden. Having better estimates of this heterogeneity, through mf count or other proxy data, and better mechanistic understanding in the origin of the heterogeneity of risk, whether through behaviour, immunity or other factors will allow us to improve failing programmes, survey for recrudescence and more accurately estimate breakpoints [26, 27]. On a short term, practical level, the use of individual data on mf, which was routinely collected in surveillance, but rarely utilised, means that models can make much more effective predictions on the impact of MDA, as illustrated in the model fitting and validation above.

The use of vector control alongside an established MDA programme has been shown to be an effective strategy in breaking transmission in high endemic areas [28]. Our results confirm this observation and suggest that fairly moderate coverages of bed-nets can lead to a dramatic decrease in the time to reach elimination. There is also the indication that vector control alone may be enough to achieve elimination in a low endemic setting. For example in the Gambia, elimination targets were achieved through the use of insecticide-treated bed-nets distributed for a malaria campaign, where there was no other LF intervention [29]. Similar observations were made in Kenya, where mf prevalence continued to decline despite missed rounds of MDA [30]. Use of bed-nets also increases the chances of elimination even after MDA has ceased [31]. We also demonstrate that the use of general vector control that target breeding sites in a *Culex* setting can also help to achieve elimination within 5 years. In these areas where malaria is co-endemic, bed-net use may be higher than the 50 % we conservatively used in our scenarios. This demonstrates the necessity to model the use of VC in order to provide more accurate estimates of elimination and also explore other mechanisms that might lead to a change in the vector dynamics such as the use of ivermectin [32].

Human behaviour and selective coverage can both have a large impact towards an elimination campaign [17, 33, 34]. We explored a number of different scenarios in which correlation between interventions and exposure lead to negative consequences for an elimination timeline. Systematic adherence was controlled for by setting the correlation between treatment rounds, where a higher correlation means an individual is more likely to choose their previous decision on whether to receive treatment. This allowed both systematic adherence as well as coverage to be altered independently in order to measure the effects of both. Systematic adherence alone has a marginal impact on the probability of elimination. However when other correlations were considered, the impact was much greater. A correlation between exposure to infective bites and non-adherence can in some cases lead to no chance of elimination where it could be achieved at high levels when this was not the case. Correlation between insecticide-treated bed-nets (LLINs) non-adherence and MDA non-adherence can also dramatically reduce probability of elimination. The other aspect to this is where MDA is more targeted towards individuals with a higher exposure. This is where individuals more highly burdened are more likely to receive treatment perhaps due to greater awareness of the disease. This can lead to a large increase in the probability of elimination. An open question is how much this correlation exists and how much it may vary between settings. Practical tools for measuring these effects would be useful for evaluating control programmes that are failing to reach their targets.

Estimation of prevalence through the use of an immuno-chromatographic card test (ICT) has rapidly become the more popular method of estimating prevalence compared to measuring mf in the peripheral blood due to its cheaper cost, easier implementation and relatively little training to perform [35]. Open questions remain in how to relate the ICT prevalence to the mf prevalence however, due to ICT measuring an antigen secreted from adult worms as opposed to measuring adult worms directly [36]. Here, we improve on previous modelling work by using the presence of adult worms in an individual as an indicator of a positive ICT and hence measuring the antigen prevalence for given scenarios. Although validation between the simulated and estimated ICT was strong in the Sri Lanka fit, it was less so in the Kenyan fit, where the ICT was consistently under-estimated. Possible reasons for this may be due to a subset of the population being infected with infertile worms that would show up under ICT and not an mf test or that antigens in the blood may remain after adult worm mortality and so ICT-positives can remain in the population even after transmission has been broken. The sensitivity and specificity of ICT and mf tests are different and this will also impact the discrepancy. Other possible mechanisms for the discrepancy between antigenaemia and microfilaraemia are due to decreasing fecundity alongside increasing heterogeneity of adult worms during an MDA campaign (Irvine M, et al. Understanding the relationship between population level prevalence of microfilariae and antigenaemia using a model of lymphatic filariasis infection, submitted). These scenarios may be modelled in the current framework in order to better estimate the relationship and provide answers for this pressing issue.

### Data & programmatic implications

The modelling work has identified a number of key areas that are important to address with regards to an elimination prgramme. In a timeline of 5 years bi-annual MDA at 65 % is the most effective of all strategies considered. However, if bi-annual MDA is not feasible, then an MDA programme combined with vector control can also have a similarly high probability of success in all settings. Annual MDA at 80 % with no VC is only effective in low and medium settings and annual MDA at 65 % is only effective for lower endemic settings. A number of systematic adherence issues can impact the success of a programme such as individuals who don’t adhere having higher burden, use of LLINs being correlated with adherence to MDA for an individual and systematic compliance to MDA. Where there are units that have not met goals within a specified timeline these would be the main sources to investigate.

How exposure is distributed among individuals due to proximity to breeding sites etc. also has large implications for the success of a programme. A proxy of this can be taken from understanding the distribution of intensity of infection. Therefore using mf or antigen count data, which is already collected, should be used to inform modelling work and threshold targets for prevalence.

Differences between Anopheles & Culex uptake curves also have important implications for elimination. Big differences in competence have also been observed for closely related species. Data on vector competence, behaviour, ecology and species distributions would allow for more accurate modelling work and aide in elimination strategies.

## Conclusion

We have considered the question of whether mass drug administration alone is capable of achieving elimination within the ambitious 2020 goals. To this end we developed a novel model of filariasis infection where an individual’s treatment, use of vector control and ability to adhere or not to interventions may be explicitly modelled. We have found that in most settings elimination may be achieved if the intervention is intensive enough. For areas with higher endemicity, this ambitious goal may not be achieved and other forms of intervention would need to be considered, principally higher coverage of both MDA and possibly additional vector control. We identified a number of issues that may inhibit an elimination campaign however. Chiefly, the aggregation of bite risk amongst a population can limit the reduction in prevalence due to MDA. Systematic adherence when coupled with exposure and adherence to other interventions can also have a large negative impact on a campaign. There is therefore a need to produce more accurate estimates of population aggregation by fully utilising individual-based data in models for elimination.

## Additional files

### Model

We introduce a novel model of filariasis transmission. The model is a stochastic micro-simulation of individuals with worm burden, microfilaraemia and other demographic parameters relating to age and risk of exposure. Humans are modelled individually, with their own male and female worm burden denoted *W*_*i*_^*m*^ and *W*_*i*_^*f*^. The density of mf in the peripheral blood is also modelled for each individual and denoted *M*_*i*_. The total mf density in the population contributes towards the current density of L3 larvae in the human-biting mosquito population. The model dynamics are divided into the individual human dynamics, including age and turnover; worm dynamics inside the host; microfilariae dynamics inside the host and larvae dynamics inside the mosquito.

### Worm dynamics

For each individual *i* both male and female worms are added according to their bite risk *b*_*i*_ that is individually drawn from a gamma distribution with mean 1 and shape parameter *k* (this means that the scale parameter is automatically determined to keep the mean of the distribution at one, hence *θ* = *k*^−1^). The rate at which worms are acquired depends on a number of stages of larvae life cycle as well as characteristics of the host. These are the mean total number of bites per individual per month *b*; the number of bites per mosquito *λ*; the ratio of vectors to hosts *V*: *H*; the probability that an L3 larvae leaves the host during a biting event *ψ*_1_; the probability that the L3 enters the host *ψ*_2_; the proportion of L3 within the host that develop into adult worms *s*_2_; and the age-dependent biting rate *h*(*a*) that increases with body size to saturate at age nine [7]. The rate at which an individual *i* acquires an adult worm is therefore

$$\frac{1}{2}\lambda {b}_i\left(V:H\right){\psi}_1{\psi}_2h(a),$$

for both male and female worms. All the parameters described can be derived from literature estimates with the exception of the bite-risk shape parameter *k* and the vector to host ratio *V:H* . Therefore both of these parameters need to be fitted for each setting. Each worm has a constant rate of death *μ*, which is the same for males and females. Therefore the number of deaths in a time-step is Poisson-distributed with rate *μW*_*i*_^*m*^ and *μW*_*i*_^*f*^ for male and female worms respectively.

### Mf dynamics

The microfilariae dynamics are dependent on the total number of adult male and female worms. *W. bancrofti* is assumed to be completely polygamous [12] and hence the rate at which mf are produced is dependent upon the number of female worms combined with the presence of male worms. It is also assumed that there is death of the microfilariae that is constant and independent of the density of the mf. The dynamics of mf for an individual *і* are therefore

$$\frac{\mathrm{d}{M}_i}{\mathrm{d}\mathrm{t}}=\alpha {W}_i^f\mathbb{I}\left({W}_i^m>0\right)\mathit{\hbox{-}}\gamma {M}_i,$$

where the function $\mathbb{I}$ is one if there are male worms and zero if not.

### Larvae dynamics

The larvae develop from the mf that enter the mosquito during a blood meal from an infected host. There are two functional forms of this relationship that differ according to mosquito genus. For *Culex*, where the cibarial armature is less-developed than in other species, mf can survive at lower densities [13]. The relationship here is

$$\mathrm{L}(m)={\kappa}_{s1}\left(1\hbox{-} {e}^{\mathit{\hbox{-}}{r}_1m/{\kappa}_{s1}}\right).$$

For *Anopheles* larvae survival and development is facilitated at higher densities of mf. This relationship is given by

$$\mathrm{L}(m)={\kappa}_{s2}{\left(1\hbox{-} {e}^{\hbox{-} {r}_2m/{\kappa}_{s2}}\right)}^2,$$

where *m* here is concentration of mf per 20 *μ*L taken during a blood meal and *r*, *κ* are parameters relating to the functional form of the uptake curve [14]. Each individual contributes towards the pool of larvae in the mosquito population according to their concentration of mf in the peripheral blood along with their intrinsic bite-risk *b*_*i*_. The uptake of mf that develop into larvae is an average of all individual’s mf concentration weighted by their bite-risk i.e.

$$\tilde{L}={\displaystyle \sum_iL\left({m}_i\right){b}_i/}{\displaystyle \sum_i{b}_i},$$

giving the average number of larvae per mosquito.

The dynamics of larvae in the mosquito are developed in very similar vain to EPIFIL [7]. The dynamics are fast compared with the other aspects of infection due to the relatively short life-span of mosquitoes compared with filarial worms. The density is dependent on the number of bites per mosquito *λ*, the proportion of mosquitoes which pick up infection when biting an infected host *ɡ*; the death rate of L3, which is dominated by mosquito death *σ*_1_ and the proportion of L3 leaving the mosquito per bite, *ψ*_1_. The average number of larvae taken up in the mosquito population $\tilde{L}$ is calculated from the uptake curves described above,

$$\frac{\mathrm{d}L}{\mathrm{d}\mathrm{t}}=\lambda g\tilde{L}\hbox{-} \left({\sigma}_1+\lambda {\psi}_1\right)L.$$

Finally the equilibrium value for L3 in a mosquito is given by

$${L}^{*}=\frac{\lambda g\tilde{L}}{\sigma_1+\lambda {\psi}_1}.$$

Hence the equilibrium value of the larvae is a scaling of the uptake of mf that develop into larvae.

### Host dynamics

Each individual begins with zero infection and a bite-rate exposure that is drawn from a Gamma-distribution with mean 1 and shape parameter *k*. The shape parameter defines how aggregated bites are amongst individuals and consequently defines the aggregation of infection amongst individuals. The human death rate *τ* is assumed to be constant throughout an individual’s lifetime with a cut-off at age 100. This results in a truncated exponential for the ages of inidividuals in a simulation.

### Coverage and adherence of MDA

Systematic adherence of individuals to mass-drug administration was modelled by assigning an individual a probability of receiving an effective dose during a treatment round *u*_*i*_ drawn from a normal distribution with mean *u*_0_ and variance *σ*^2^,

$${u}_i\sim N\left({u}_0,{\sigma}^2\right).$$

During each treatment round an individual is treated according to whether a randomly assigned variable *z* is less than zero. This random variable is drawn from a normal distribution with mean *u*_*i*_ and variance 1.

$$z\sim N\left({u}_i,1\right).$$

For there to be a coverage of *p*, the following must hold,

$$p=\varPhi \left(\mathit{\hbox{-}}{u}_0/\sqrt{1+{\sigma}^2}\right),$$

where *Φ* is the standard normal cumulative distribution function. The value for *u*_0_ may therefore be calculated using a pre-defined treatment coverage as

$${u}_0=\hbox{-} {\Phi}^{\hbox{-} 1}(p)\sqrt{1+{\sigma}^2}.$$

The amount of systematic adherance is controlled through the variance *σ*^2^, which is defined as

$${\sigma}^2=\frac{\rho }{1\hbox{-} \rho }.$$

For *ρ =* 0 the variance is also zero and hence the receiving treatment is uncorrelated between individuals. This would be when there is no systematic adherance. As *ρ* increases to 1, the variance for an individual’s random choice value *u*_*i*_ increases meaning it is more likely to be far above or below zero and hence means that adherence is more strongly correlated for an individual. This provides a method for changing the amount of systematic adherence without altering the total treatment coverage.

We simulated a number of scenarios of intervention alongside potential complexities which may limit the effectiveness of the intervention. The initial group of scenarios studied are for MDA coverage at 65, 80 and 95 % along with systematic adherence levels of 0.0, where each round of treatment has random coverage which is not correlated with previous rounds; 0.5, where an individual’s decision to comply is the same as in the previous round 50 % of the time and 1.0, where an individual either always complies or never complies with MDA. These scenarios are considered for low, medium and high endemic settings and for Anophelene and Culicine dominated regions.

For the annual MDA setting with the *Anopheles* vector, coverage rapidly speeds up the time to elimination in low and medium settings (Additional file 1: Figure S1). The probability of elimination to reach 50 % in a low setting reduces from approximately 8 to 5 years as coverage increases. The medium setting is more pronounced reducing the time from 15 to 6 years. Annual MDA alone is not enough to achieve elimination in all but the highest coverage of MDA, where the time to achieve elimination in 50 % of runs is approximately 15 years. The effect of systematic adherence generally increases the time to elimination, however, this effect is relatively small compared with the effect of increased coverage, increasing the 50 % elimination probability time by at most 2 years for all cases, except in the medium endemic setting in low coverage where there is an increase by 5 years.

For an annual MDA programme in a Culicine region, annual MDA is ineffective for medium and high endemic settings at low to medium coverage (Additional file 2: Figure S2). For low endemicity, the time to elimination is dramatically increased compared to an Anophelene region and the time to elimination is less than a decade for only the highest coverage. Systematic adherence again has a marginal effect on the time to elimination.

Increasing from annual to bi-annual MDA significantly decreases the time to elimination for low and medium endemic settings as well as being able to break transmission for the highest endemic level (Additional file 3: Figure S3). High coverage can reduce the time to elimination to under 5 years for medium and high coverage, with systematic adherence only have a marginal effect on the outcome of the campaign.

Finally for bi-annual MDA in an Anophelene region, elimination can be achieved within 5 years for all endemic settings and levels of systematic adherence (Additional file 4: Figure S4). In a low endemic setting 65 % coverage is enough to achieve elimination within 5 years, whilst 80 % coverage is required for a medium setting. To achieve elimination within 5 years for a high setting, a coverage of 95 % is required and this is not altered by the presence of systematic adherence.

### Correlation between exposure and tendency to comply with MDA

The presence of correlation between exposure and tendency to comply with MDA was modelled for various coverages and strengths of correlation. This was implemented by first constructing a multi-variate normal distribution with the following covariate matrix

$$ \varSigma =\left(\begin{array}{cc}\hfill {\sigma}^2\hfill & \hfill \sigma {\rho}_{BC}\hfill \\ {}\hfill \sigma {\rho}_{BC}\hfill & \hfill 1\hfill \end{array}\right), $$

where *ρ*_*BC*_ is the correlation between the bite-exposure and the systematic adherence of an individual and can vary between −1 and 1. The next step is to construct the mean of the multivariate normal, defined as

$$M={\left({u}_0,0\right)}^T.$$

An individual’s tendency to comply with MDA *u*_*i*_ and a standard normal variable representing their bite risk (${\widehat{b}}_i$) are drawn from a multivariate normal distribution as

$$\left({u}_i,{\widehat{b}}_i\right)\sim \mathrm{M}\mathrm{V}\mathrm{N}\left(M,\varSigma \right).$$

In order to convert the normal-distributed bite-risk${\widehat{b}}_i$ into the gamma-distributed bite-risk *b*_*i*_, which is used in the simulations, the inverse cumulative distribution function (CDF) of the standard normal is first applied converting into a uniformly distributed random number. The inverse of the gamma CDF is then applied to this random variable, producing a gamma distributed random variable that is still correlated with the tendency to comply or not comply with MDA.

The probability of achieving elimination was explored if there is a positive, or negative correlation between an individual’s bite risk and their adherence with an MDA programme (Additional file 5: Figure S5 & Additional file 6: Figure S6). MDA was performed bi-annually for 5 years at three coverage levels and four levels of systematic adherence from none, weak, strong and very strong. The correlation greatly affects the outcome of an MDA programme in a short time-frame and dramatically increases the probability of elimination at low coverage levels.

In the low endemic setting, coverage of 80 % is enough to achieve elimination within 5 years with probability over 90 %, when there is no systematic adherence (Additional file 5: Figure S5). When adherence is linked to bite risk there is a severe reduction in probability of elimination at low and medium coverages. The same level of reduction is not observed in the highest coverage with only a marginal decrease of 20 % points in the most severe case.

For a high endemic setting, a correlation between bite-risk can decrease or increase the probability of elimination depending on whether exposure is linked to adherence or non-adherence. When there is a link between exposure and adherence, a moderate coverage of 80 % can increase the probability of elimination from 0 to 45 %. A correlation between exposure and non-adherence by contrast severely reduces the probability of elimination from over 60 % for high coverage of MDA to less than 10 % (Additional file 6: Figure S6).

### Vector control

Vector control was again modelled for low, medium and high endemic settings defined as average endemic prevalence of 10, 15 and 20 %. The individual-based model of long-lasting insecticide treated bed nets (LLINs) were modelled for coverages of 65, 80 and 95 %. These were assumed to not be correlated with the MDA programme, which had coverage of 65 % with both annual and bi-annual interventions modelled.

The probability of elimination was measured for each setting, LLIN coverage and annual and bi-annual MDA. For the annual intervention setting, bed net coverage alters the probability to elimination from the baseline (Additional file 7: Figure S7). Moderate coverage of 65 % is enough to achieve 100 % elimination within 5 years in a low endemic setting, whilst also increasing the probability in a high endemic setting from zero to 10 %. Increasing the bed net coverage to 95 % of the population also dramatically increases the probability of elimination with a high coverage of MDA leading the 100 % probability of elimination in all but the high endemic setting categories.

The impact of the use of LLINs on a bi-annual MDA programme is also pronounced (Additional file 8: Figure S8). For moderate coverage of 65 % along with moderate coverage of MDA the probability of elimination is 100, 90 and 20 % for low,medium and high settings respectively. This is a dramatic increase in the elimination probability from an annual campaign where even high bed net coverage is not enough to achieve significant probability for a moderate coverage of MDA. For high bi-annual coverage of MDA, elimination is achieved in all endemic settings. Vector control combined with a bi-annual elimination programme is therefore able to eliminate within 5 years for a large range of endemicity.

### Correlation between adherence of MDA and VC

Positive correlation between LLIN and MDA adherence was considered for different systematic adherence levels and coverage of LLIN (Additional file 9: Figure S9). A 65 % coverage annual MDA campaign was implemented alongside a bed-net campaign that are distributed during the first round of MDA. Weak to strong systematic adherence combined with a correlation between LLIN and MDA can negatively impact the outcome of the intervention when coverage of bed nets is low. This is due to individuals who are treated are also more likely to use bed-nets and so a larger proportion of the population receive neither intervention. When coverage of bed nets is high, elimination occurs in nearly all cases and so this correlation matters less. However, when coverage is low and there is a high probability of not achieving elimination for the same conditions, the correlation between interventions can reduce the probability of elimination by as much as 60 %.

### Impact of these correlations on the dynamics

In order to investigate the effect of these different correlations on the dynamics of an MDA programme, we consider a range of systematic adherence parameters (*ρ* = 0.0,0.5,0.99) and a range of coverage levels (*p*_C_ = 0.65,0.8,0.95) for high medium and low prevalence (10, 15 and 20 % mf prevalence). We compare the reductions in prevalence 5 years into the programme probability of elimination for each of these settings, where elimination is defined as when transmission has been broken and no bounce back to pre-intervention levels can occur, even when intervention has been stopped. Systematic adherence alone has a marginal impact on the outcome of a MDA programme (Additional file 1: Figure S1, Additional file 2: Figure S2, Additional file 4: Figure S4, Additional file 3: Figure S3 Other complications however, have a larger impact on the effectiveness of an intervention programme.

For annual MDA at 65 %, coverage of 65 % is only able to achieve elimination after 5 years for a low endemicity and after 10 years for medium endemicity (Additional file 1: Figure S1). 65 % is not enough to achieve elimination in the high endemic setting. Increasing to 80 % coverage shortens the time to elimination for low and medium endemicity and is able to achieve elimination in 10 % of cases after 15–20 years of MDA. High coverage of 95 % rapidly speeds up the time to elimination for low and medium endemic settings to between 4 and 8 years. The probability of elimination in a high endemic setting begins to increase around 9 years of MDA and continues to climb to 60 % after 20 years of annual MDA.

### Calculating the vector to human ratio, prevalence relationship

In order to simulate a number of diverse scenarios, we required simulations to have endemic states at a number of different prevalences. As prevalence is not a free parameter in the model, this was done by changing the vector to human ratio *V*: *H*. We therefore calculated the mean prevalence over 200 simulation runs or a range of vector to human ratios and used the resulting relationship to calculate the required parameter for a given prevalence value (Additional file 10: Figure S10). The relationship was also calculated for the two main vector genera *Anopheles* and *Culex*. For a large vector to host ratio there is no significant difference in prevalence, however a lower ratios there is an appreciable difference, where *Culex* has a breakpoint lower than *Anopheles*. For a range of values, high, medium and low transmission settings were required. The prevalences taken were 10, 15 and 20 % with the corresponding *V*: *H* estimated by using the first value for which that prevalence is achieved. These were estimated as 39, 60 and 120.

## Additional files

Additional file 1: Figure S1. Elimination timeline for annual MDA in *Anopheles* setting. Scenario simula tions for probability to elimination in annual treatment for *Anopheles* genus at different coverages and systematic adherence levels. (PDF 368 kb) Additional file 2: Figure S2. Elimination timeline for annual MDA in *Culex* setting. Scenario simulations for probability to elimination in annual treatment for *Culex* genus at different coverages and systematic adherence levels. (PDF 387 kb) Additional file 3: Figure S3. Elimination timeline for bi-annual MDA in *Culex* setting. Scenario simulations for probability to elimination in bi-annual treatment for *Culex* genus at different coverages and systematic adherence levels. (PDF 369 kb) Additional file 4: Figure S4. Elimination timeline for bi-annual MDA in *Anopheles* setting. Scenario simulations for probability to elimination in bi-annual treatment for *Anopheles* genus at different coverages and systematic adherence levels. (PDF 367 kb) Additional file 5: Figure S5. Impact of correlation between bite risk and adherence to MDA in low setting . Probability of elimination after 5 years for an annual MDA programme at 65 % coverage in a low endemic setting where there is either a negative, none or positive correlation between the bite-risk of an individual and their tendency to comply with an MDA programme. The underlying endemicity considered here is low. (PDF 262 kb) Additional file 6: Figure S6. Impact of correlation between bite risk and adherence to MDA in high setting. Probability of elimination after 5 years for an annual MDA programme at 65 % coverage in a high endemic setting, where there is either a negative, no or positive correlation between the bite-risk of an individual and their tendency to comply with an MDA programme. Here the underlying endemicity is high. (PDF 267 kb) Additional file 7: Figure S7. Elimination probability for annual MDA with VC. Simulations were performed to calculate the probability to elimination within 5 years annual treatment combined with vector control of *A. gambiae*. (a) bed-net coverage 65 %, (b) bed-net coverage 80 % and (c) bed-net coverage 95 %. (PDF 160 kb) Additional file 8: Figure S8. Elimination probability for bi-annual MDA with VC. Simulations were per- formed to calculate the probability to elimination within 5 years for bi-annual treatment combined with vector control of *A. gambiae*. (a) bed-net coverage 65 %, (b) bed-net coverage 80 % and (c) bed-net coverage 95 %. (PDF 159 kb) Additional file 9: Figure S9. Impact of correlation between LLIN and MDA adherence. Correlation between LLIN coverage and MDA adherence is shown for different systematic adherence levels and coverage of LLIN. A 65 % coverage annual MDA campaign was implemented alongside a bed-net campaign that is distributed during the first round of MDA. Systematic adherences were simulated for (a) none, (b) weak, (c) strong and (d) very strong. Weak to strong systematic adherence combined with a correlation between LLIN and MDA can negatively impact the outcome of the intervention. (PDF 246 kb) Additional file 10: Figure S10. Relationship between vector to host ratio & prevalence. Calculated relationship between the vector to human ratio and the mf prevalence for *Anopheles & Culex* in the model for 200 simulation runs for each point. Variability of the prevalence is displayed as error bars for the standard deviation of the endemic prevalence of at each vector to host ratio. (PDF 104 kb)
