# Supplementary material for: Modelling strategies to break transmission of lymphatic filariasis - aggregation, adherence and vector competence greatly alter elimination
Source: Parasit Vectors. 2015 Oct 22;8:547. doi: 10.1186/s13071-015-1152-3 (PMC4618540; doi:10.1186/s13071-015-1152-3)
Supplement: Additional file 1: Figure S1. — Elimination timeline for annual MDA in Anopheles setting. Scenario simula tions for probability to elimination in annual treatment for Anopheles genus at different coverages and systematic adherence levels. (PDF 368 kb) [file 13071_2015_1152_MOESM1_ESM.pdf]

Coverage/ adherence	0.0	0.5	1.0
65%			
80%			
95%			

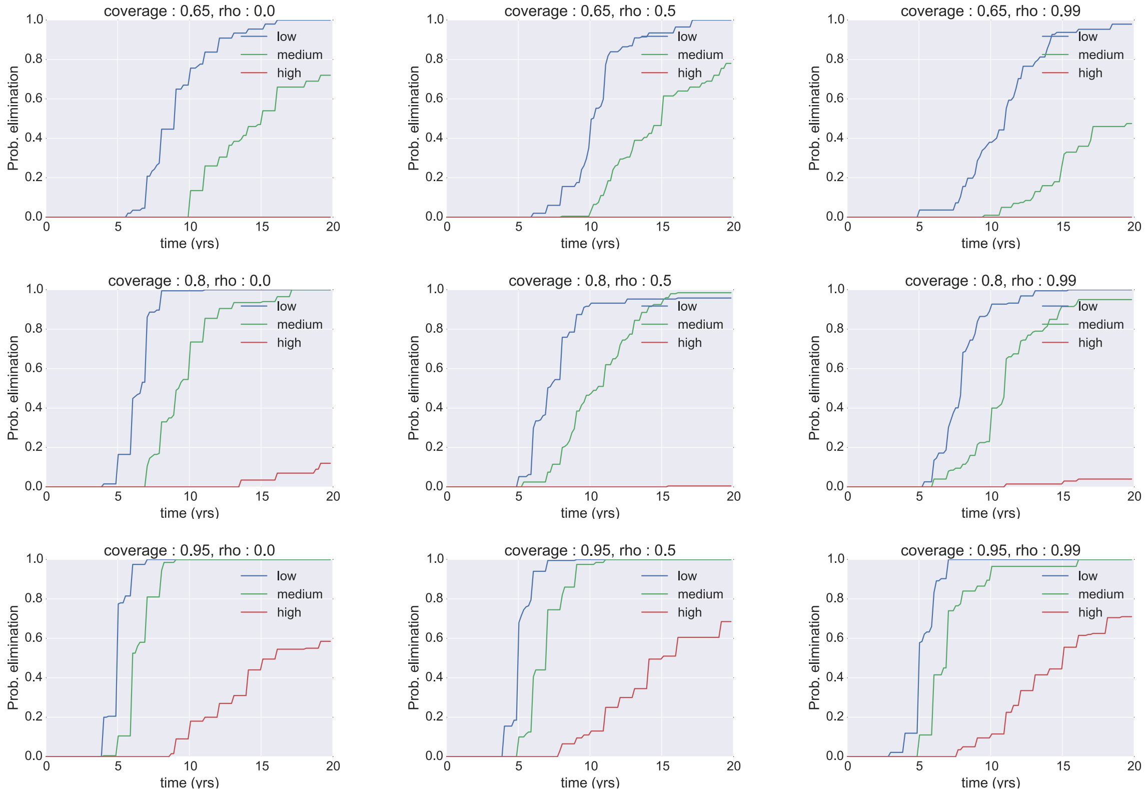

Figure 9: **Elimination timeline for annual MDA in *Anopheles* setting**. Scenario simulations for probability to elimination in annual treatment for *Anopheles* genus at different coverages and systematic adherence levels.
