# Supplementary material for: Modelling strategies to break transmission of lymphatic filariasis - aggregation, adherence and vector competence greatly alter elimination
Source: Parasit Vectors. 2015 Oct 22;8:547. doi: 10.1186/s13071-015-1152-3 (PMC4618540; doi:10.1186/s13071-015-1152-3)
Supplement: Additional file 2: Figure S2. — Elimination timeline for annual MDA in Culex setting. Scenario simulations for probability to elimination in annual treatment for Culex genus at different coverages and systematic adherence levels. (PDF 387 kb) [file 13071_2015_1152_MOESM2_ESM.pdf]

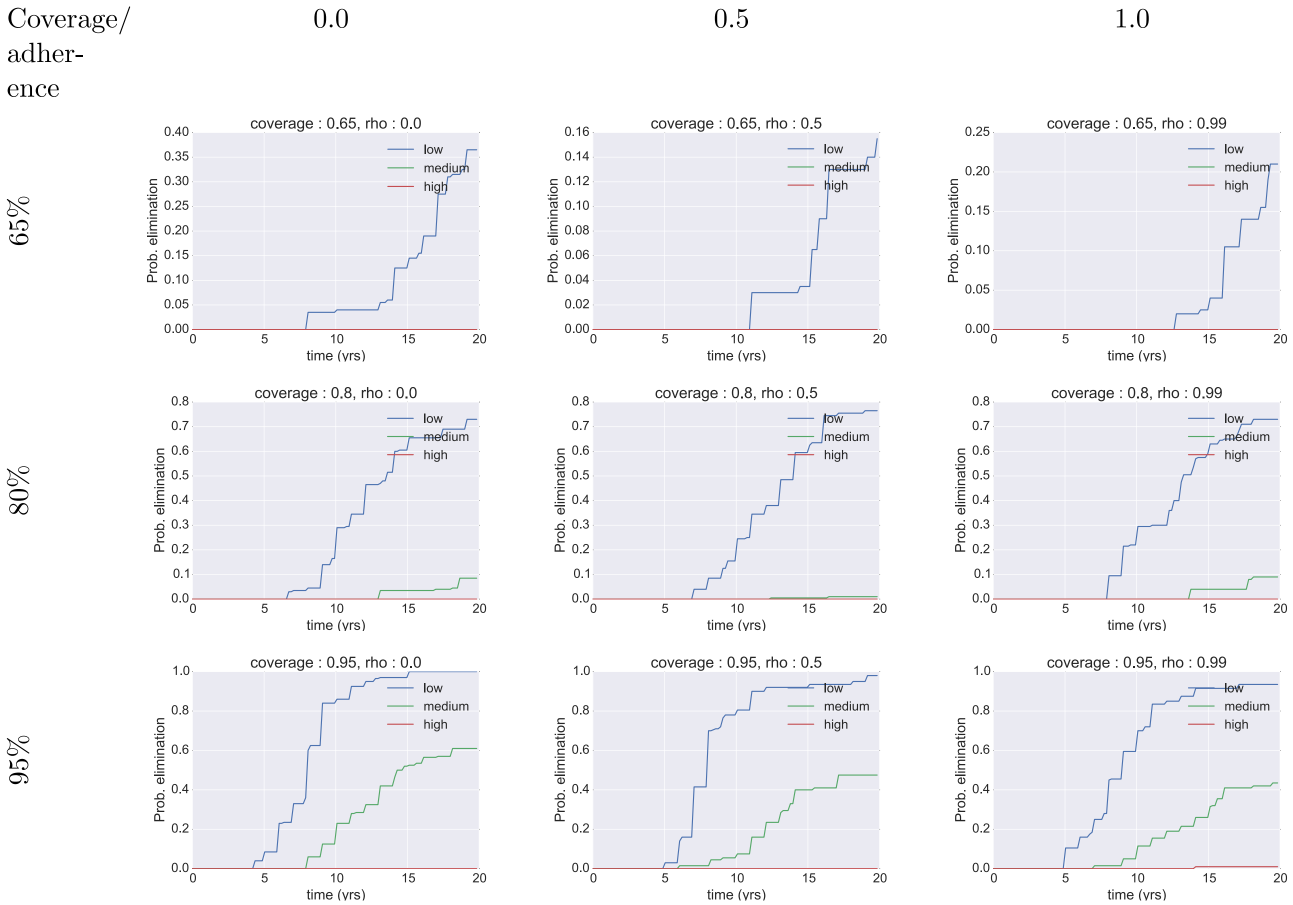

Figure 10: **Elimination timeline for annual MDA in *Culex* setting**. Scenario simulations for probability to elimination in annual treatment for *Culex* genus at different coverages and systematic adherence levels.
