# Supplementary material for: Modelling strategies to break transmission of lymphatic filariasis - aggregation, adherence and vector competence greatly alter elimination
Source: Parasit Vectors. 2015 Oct 22;8:547. doi: 10.1186/s13071-015-1152-3 (PMC4618540; doi:10.1186/s13071-015-1152-3)
Supplement: Additional file 3: Figure S3. — Elimination timeline for bi-annual MDA in Culex setting. Scenario simulations for probability to elimination in bi-annual treatment for Culex genus at different coverages and systematic adherence levels. (PDF 369 kb) [file 13071_2015_1152_MOESM3_ESM.pdf]

Coverage/ adherence	0.0	0.5	1.0
65%	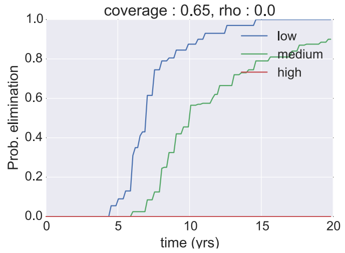	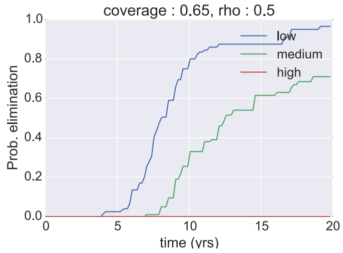	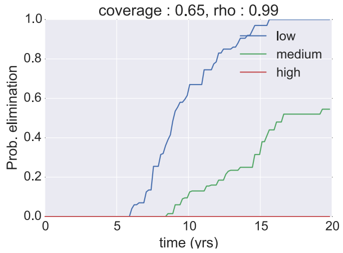
80%	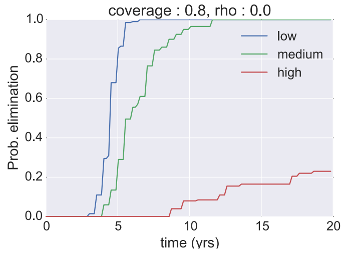	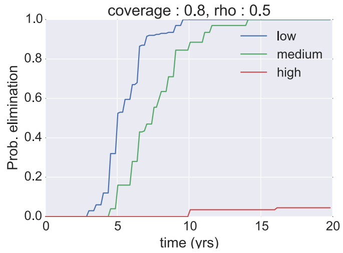	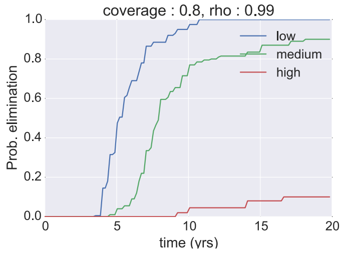
95%	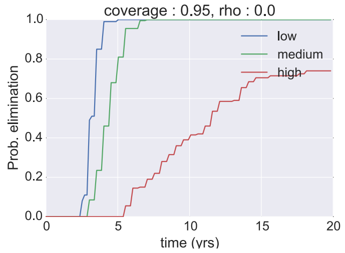	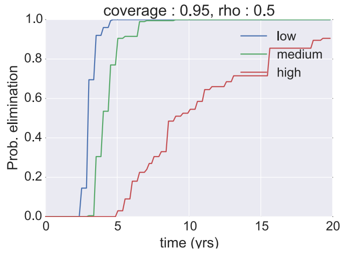	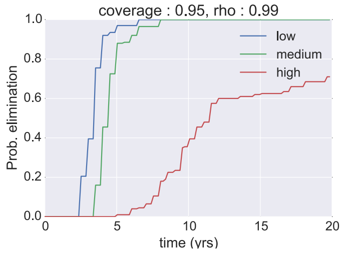

Figure 12: **Elimination timeline for bi-annual MDA in *Culex* setting** . Scenario simulations for probability to elimination in bi-annual treatment for *Culex* genus at different coverages and systematic adherence levels.
