# Supplementary material for: Modelling strategies to break transmission of lymphatic filariasis - aggregation, adherence and vector competence greatly alter elimination
Source: Parasit Vectors. 2015 Oct 22;8:547. doi: 10.1186/s13071-015-1152-3 (PMC4618540; doi:10.1186/s13071-015-1152-3)
Supplement: Additional file 4: Figure S4. — Elimination timeline for bi-annual MDA in Anopheles setting. Scenario simulations for probability to elimination in bi-annual treatment for Anopheles genus at different coverages and systematic adherence levels. (PDF 367 kb) [file 13071_2015_1152_MOESM4_ESM.pdf]

Coverage/ adherence | 0.0 | 0.5 | 1.0

65% | 80% | 95%

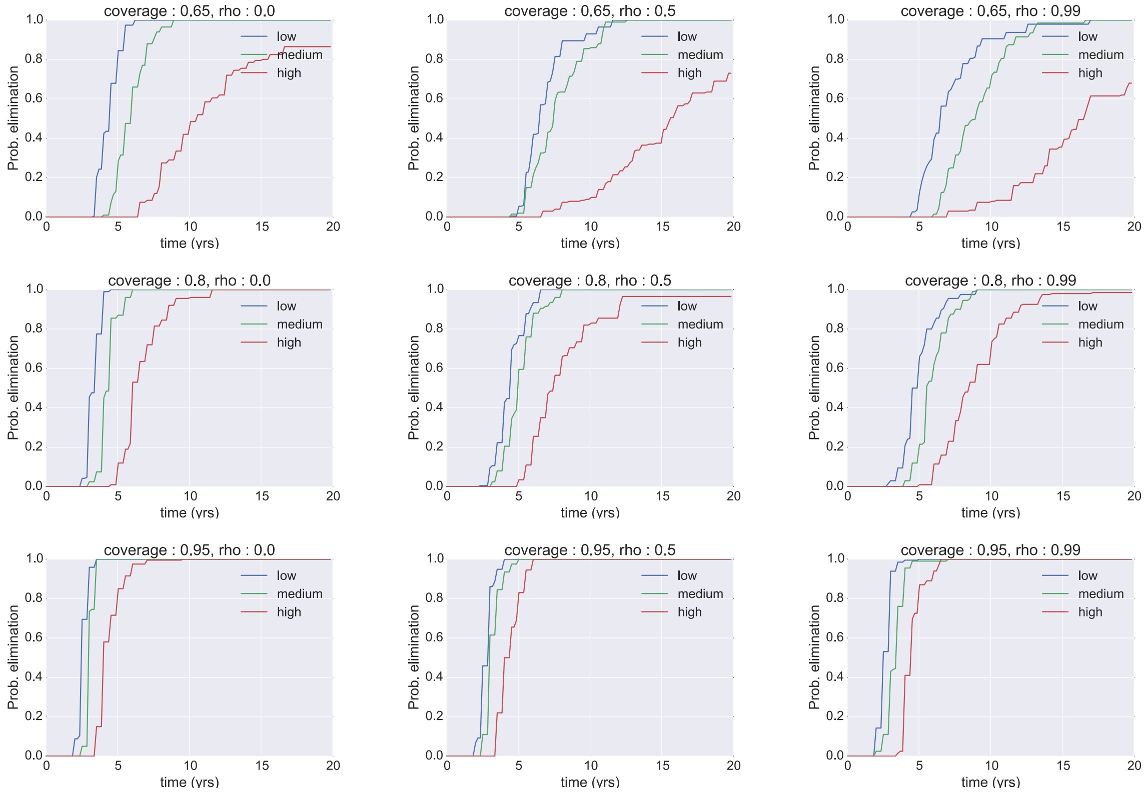

Figure 11: **Elimination timeline for bi-annual MDA in *Anopheles* setting**. Scenario simulations for probability to elimination in bi-annual treatment for *Anopheles* genus at different coverages and systematic adherence levels.
