# Supplementary material for: Modelling strategies to break transmission of lymphatic filariasis - aggregation, adherence and vector competence greatly alter elimination
Source: Parasit Vectors. 2015 Oct 22;8:547. doi: 10.1186/s13071-015-1152-3 (PMC4618540; doi:10.1186/s13071-015-1152-3)
Supplement: Additional file 5: Figure S5. — Impact of correlation between bite risk and adherence to MDA in low setting . Probability of elimination after 5 years for an annual MDA programme at 65 % coverage in a low endemic setting where there is either a negative, none or positive correlation between the bite-risk of an individual and their tendency to comply with an MDA programme. The underlying endemicity considered here is low. (PDF 262 kb) [file 13071_2015_1152_MOESM5_ESM.pdf]

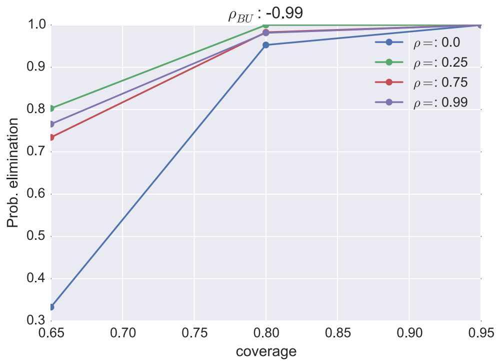

(a)

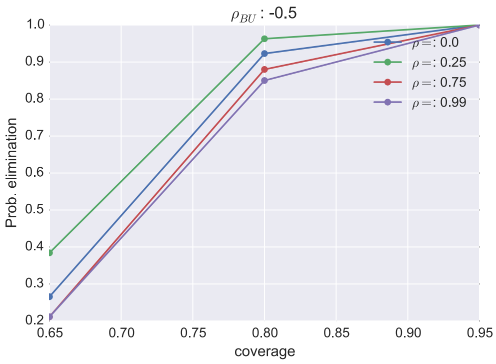

(b)

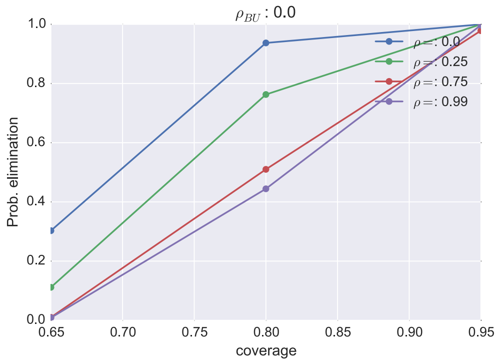

(c)

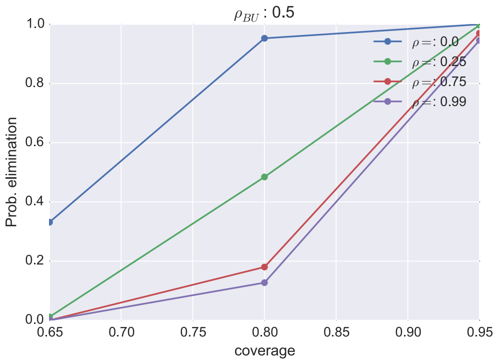

(d)

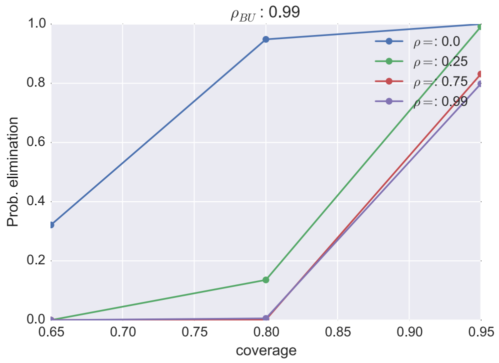

(e)

Figure 13: **Impact of correlation between bite risk and adherence to MDA in low setting** . Probability of elimination after five years for an annual MDA programme at 65% coverage in a low endemic setting where there is either a negative, none or positive correlation between the bite-risk of an individual and their tendency to comply with an MDA programme. The underlying endemicity considered here is low.
