# Supplementary material for: Modelling strategies to break transmission of lymphatic filariasis - aggregation, adherence and vector competence greatly alter elimination
Source: Parasit Vectors. 2015 Oct 22;8:547. doi: 10.1186/s13071-015-1152-3 (PMC4618540; doi:10.1186/s13071-015-1152-3)
Supplement: Additional file 6: Figure S6. — Impact of correlation between bite risk and adherence to MDA in high setting. Probability of elimination after 5 years for an annual MDA programme at 65 % coverage in a high endemic setting, where there is either a negative, no or positive correlation between the bite-risk of an individual and their tendency to comply with an MDA programme. Here the underlying endemicity is high. (PDF 267 kb) [file 13071_2015_1152_MOESM6_ESM.pdf]

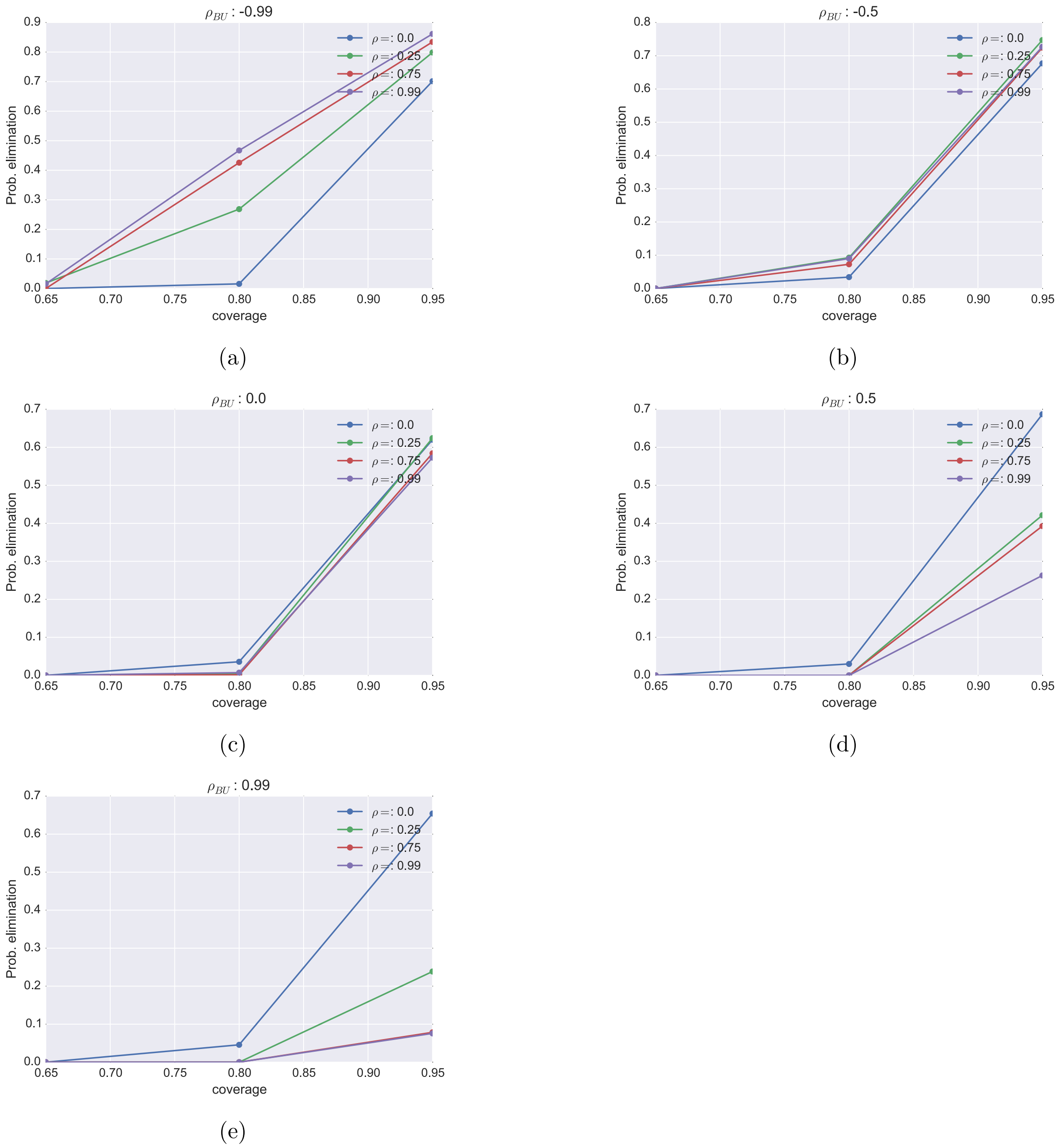

Figure 14: **Impact of correlation between bite risk and adherence to MDA in high setting**. Probability of elimination after five years for an annual MDA programme at 65% coverage in a high endemic setting, where there is either a negative, no or positive correlation between the bite-risk of an individual and their tendency to comply with an MDA programme. Here the underlying endemicity is high.
