# Supplementary material for: Modelling strategies to break transmission of lymphatic filariasis - aggregation, adherence and vector competence greatly alter elimination
Source: Parasit Vectors. 2015 Oct 22;8:547. doi: 10.1186/s13071-015-1152-3 (PMC4618540; doi:10.1186/s13071-015-1152-3)
Supplement: Additional file 7: Figure S7. — Elimination probability for annual MDA with VC. Simulations were performed to calculate the probability to elimination within 5 years annual treatment combined with vector control of A. gambiae. (a) bed-net coverage 65 %, (b) bed-net coverage 80 % and (c) bed-net coverage 95 %. (PDF 160 kb) [file 13071_2015_1152_MOESM7_ESM.pdf]

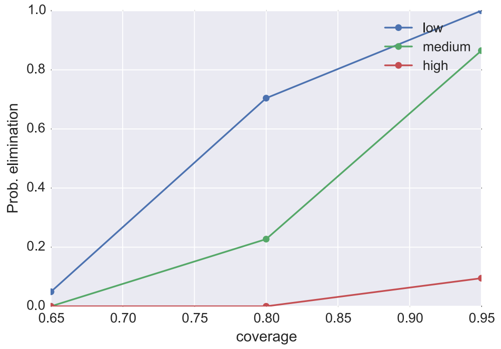

(a)

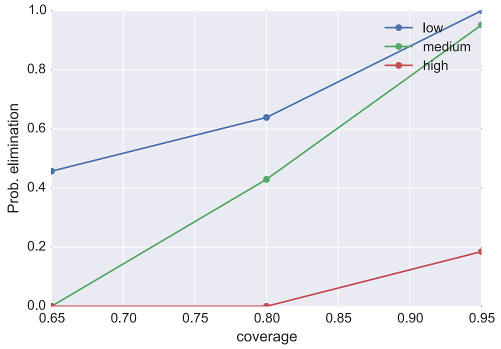

(b)

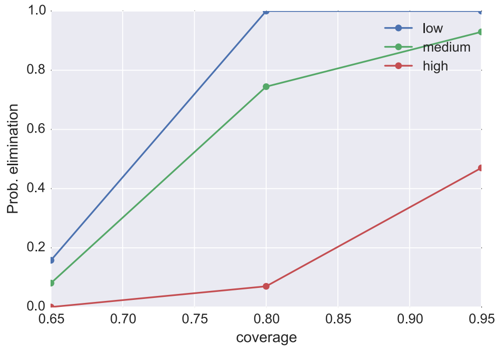

(c)

Figure 17: **Elimination probability for annual MDA with VC**. Simulations were performed to calculate the probability to elimination within five years annual treatment combined with vector control of *A. gambiae*. **(a)** bed-net coverage 65%, **(b)** bed-net coverage 80% and **(c)** bed-net coverage 95%
