# Supplementary material for: Modelling strategies to break transmission of lymphatic filariasis - aggregation, adherence and vector competence greatly alter elimination
Source: Parasit Vectors. 2015 Oct 22;8:547. doi: 10.1186/s13071-015-1152-3 (PMC4618540; doi:10.1186/s13071-015-1152-3)
Supplement: Additional file 9: Figure S9. — Impact of correlation between LLIN and MDA adherence. Correlation between LLIN coverage and MDA adherence is shown for different systematic adherence levels and coverage of LLIN. A 65 % coverage annual MDA campaign was implemented alongside a bed-net campaign that is distributed during the first round of MDA. Systematic adherences were simulated for (a) none, (b) weak, (c) strong and (d) very strong. Weak to strong systematic adherence combined with a correlation between LLIN and MDA can negatively impact the outcome of the intervention. (PDF 246 kb) [file 13071_2015_1152_MOESM9_ESM.pdf]

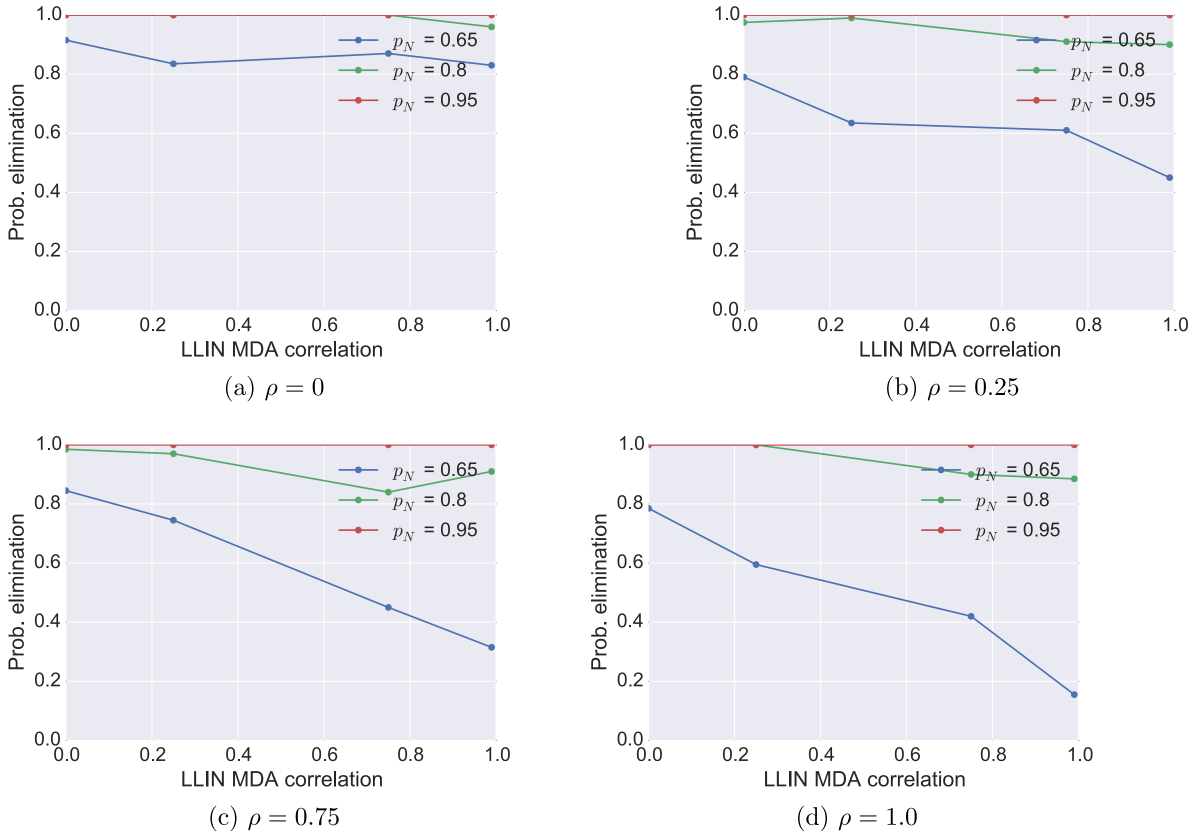

Figure 16: **Impact of correlation between LLIN and MDA adherence**. Correlation between LLIN coverage and MDA adherence is shown for different systematic adherence levels and coverage of LLIN. A 65% coverage annual MDA campaign was implemented alongside a bed-net campaign that are distributed during the first round of MDA. Systematic adherences were simulated for **(a)** none, **(b)** weak, **(c)** strong and **(d)** very strong. Weak to strong systematic adherence combined with a correlation between LLIN and MDA can negatively impact the outcome of the intervention.
