# Supplementary material for: Modelling strategies to break transmission of lymphatic filariasis - aggregation, adherence and vector competence greatly alter elimination
Source: Parasit Vectors. 2015 Oct 22;8:547. doi: 10.1186/s13071-015-1152-3 (PMC4618540; doi:10.1186/s13071-015-1152-3)
Supplement: Additional file 10: Figure S10. — Relationship between vector to host ratio & prevalence. Calculated relationship between the vector to human ratio and the mf prevalence for Anopheles & Culex in the model for 200 simulation runs for each point. Variability of the prevalence is displayed as error bars for the standard deviation of the endemic prevalence of at each vector to host ratio. (PDF 104 kb) [file 13071_2015_1152_MOESM10_ESM.pdf]

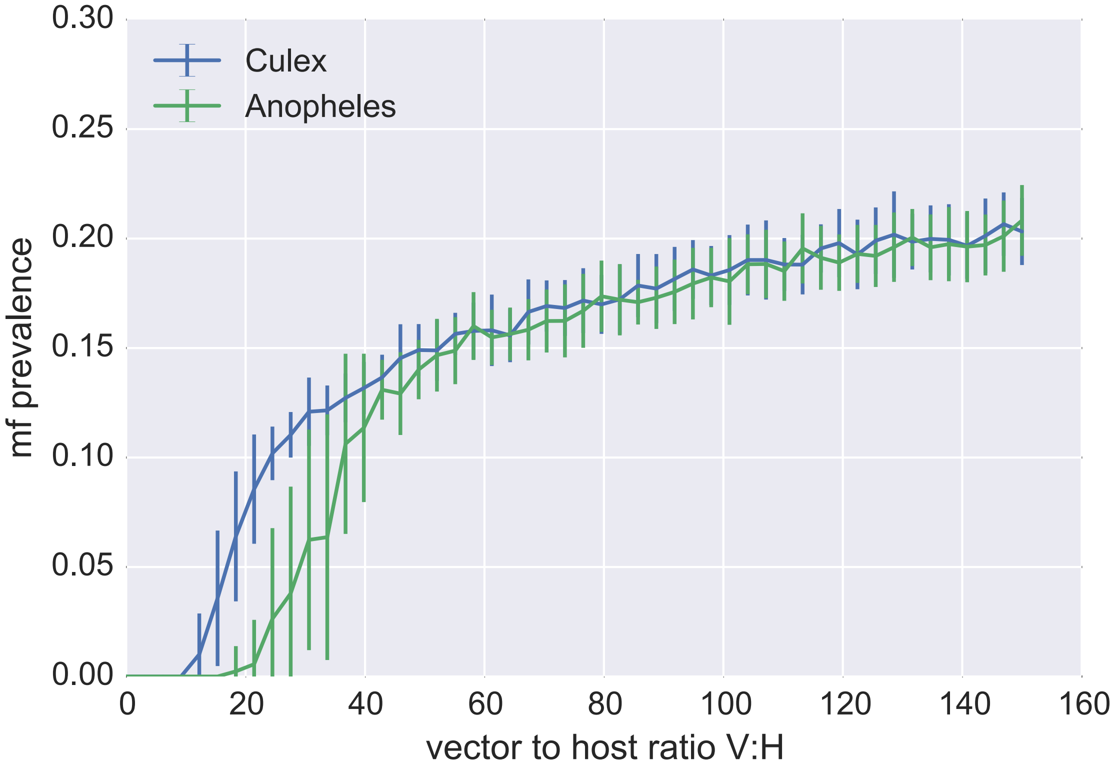

Figure 15: **Relationship between vector to host ratio & prevalence**. Calculated relationship between the vector to human ratio and the mf prevalence for *Anopheles* & *Culex* in the model for 200 simulation runs for each point. Variability of the prevalence is displayed as error bars for the standard deviation of the endemic prevalence of at each vector to host ratio.
